# Hemodynamic Effects of Stent-Induced Straightening of Parent Artery vs. Stent Struts for Intracranial Bifurcation Aneurysms

**DOI:** 10.3389/fneur.2021.802413

**Published:** 2022-02-08

**Authors:** Hailin Wan, Gang Lu, Liang Ge, Lei Huang, Yeqing Jiang, Xiaochang Leng, Jianping Xiang, Xiaolong Zhang

**Affiliations:** ^1^Huashan Hospital, Fudan University, Shanghai, China; ^2^ArteryFlow Technology Co., Ltd, Hangzhou, China

**Keywords:** stent, straightening, hemodynamics, intracranial bifurcation aneurysms, finite element method

## Abstract

**Objective:**

This study aims to compare the hemodynamic impact of stent-mesh and stent-induced straightening of the parent artery in intracranial bifurcation aneurysms using finite element method simulation.

**Material and Methods:**

Three intracranial bifurcation aneurysms treated with different stent-assisted coil embolization were evaluated. Simulation using the finite element method was conducted for Solitaire, LVIS and Neuroform stents. Four models of each stent were established, including a pre-treatment baseline, stenting without parent artery straightening (presented as stent-mesh effect), no-stent with parent artery reconstruction (to reveal the straightening impact), and stenting with straightening (categorized as Models I–IV respectively). Hemodynamic characteristics of the four models for each stent were compared.

**Results:**

In the Neuroform stent, compared with the pre-treatment model (100%), the mean WSS decreased to 82.3, 71.4, and 57.0% in Models II-IV, velocity to 88.3, 74.4, and 62.8%, and high flow volume (HFV, >0.3 m/s) to 77.7, 44.0, and 19.1%. For the LVIS stent, the mean WSS changed to 105.0, 40.2, and 39.8% in Models II to IV; velocity to 91.2, 58.1, and 52.5%, and HFV to 92.0, 56.1, and 43.9%. For the Solitaire stent, compared with the pre-treatment model (100%), the mean WSS of Models II-IV changed altered by 105.7, 42.6, and 39.4%, sac-averaged velocity changed to 111.3, 46.6, and 42.8%, and HFV 115.6, 15.1, and 13.6%.

**Conclusion:**

The hemodynamic effect of straightening the parent artery of intracranial bifurcation aneurysms by stenting was noticeably improved over stent mesh diversion in all three stents tested. Therefore stent-induced remodeling of the parent artery appears to be the best method of decreasing recurrence in intracranial bifurcation aneurysms.

## Introduction

Stent-assisted coil embolization can decrease the recurrence rate compared with simple coiling in intracranial aneurysms (1). However, the stent has a scaffold function and can induce angular deformation of parent arteries in the intracranial bifurcation aneurysms (2–4). It can migrate the flow impingement away from the aneurysm neck to decrease recurrence (5–7). Meanwhile, a stent with its mesh has a flow diverter effect by reducing the WSS and velocity of the aneurysm sac (8). However, the hemodynamic impact of the stent mesh vs. stent-induced straightening of the parent artery of the intracranial bifurcation aneurysm is unknown. This study aims to assess and compare the hemodynamic characteristics of stent-meshes and stent-induced straightening of parent artery in intracranial bifurcation aneurysms based on computational simulations using the high-fidelity finite element method.

## Materials and Methods

### Study Design

**Figure 1:**
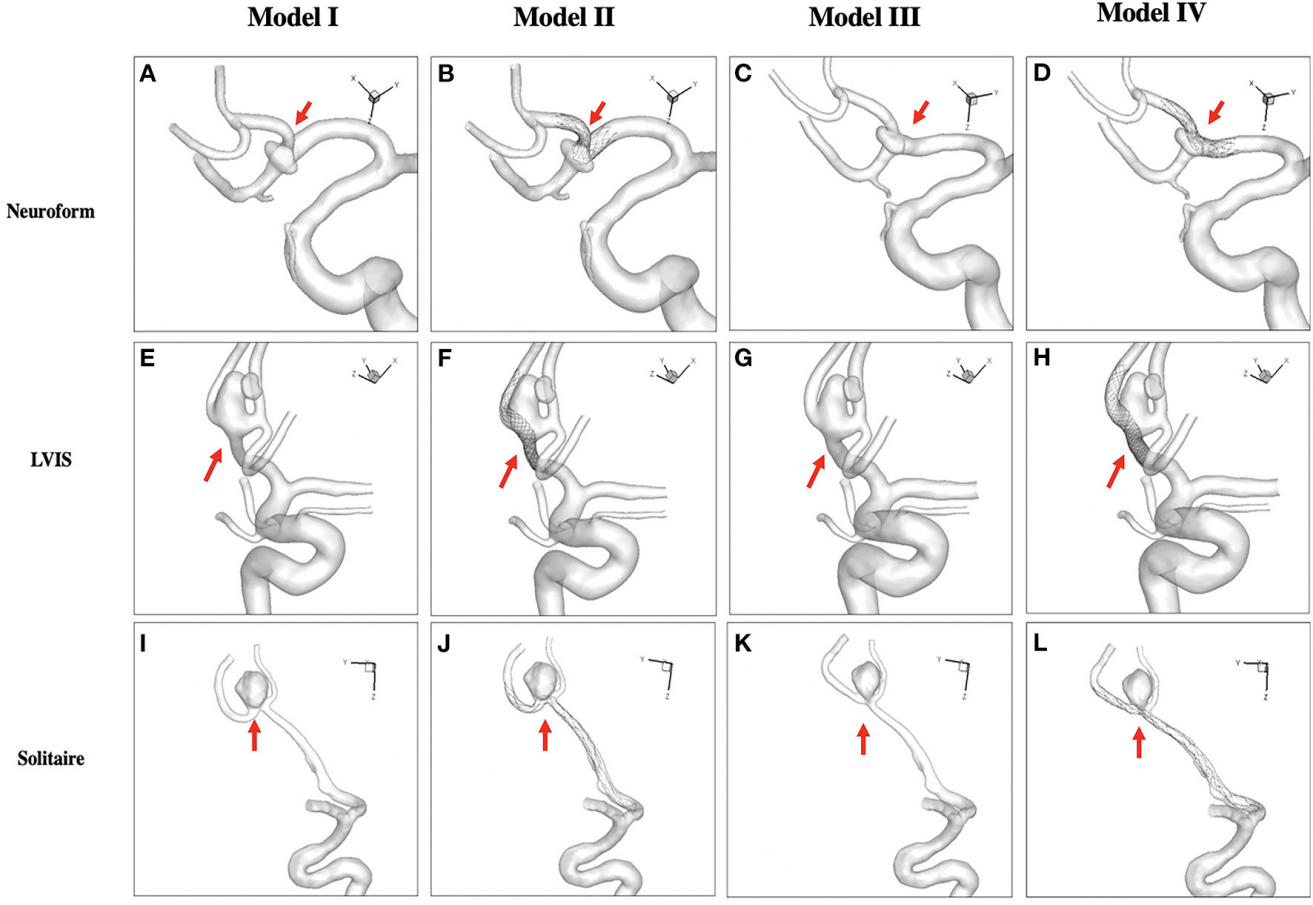
Four models of each stent (Neuroform stent, **A-D**; LVIS stent, **E-H**; Solitaire stent, **I-L**) were simulated. Pre-treatment (Model I, **A,E,I**), stenting without straightening (Model II, **B,F,J**) presented as the stent-mesh effect, no-stent with reconstruction (Model III, **C,G,K**) to reveal the straightening effect, and stenting with straightening (Model IV, **D,H,L**) were established.

Three intracranial bifurcation aneurysms treated with three different stent-assisted coil embolization were evaluated. Stenting methods including Solitaire, LVIS, and Neuroform were simulated using the finite element method. Four models of each stent, including pre-treatment (Model I), stenting without parent artery deformation (Model II, presented as stent-mesh effect), no-stent with parent artery reconstruction (model III) to reveal the straightening effect, and stenting with straightening (model IV) were established (Figure 1). Hemodynamic characteristics of the four models in each stent were compared.

### Patient Description and Aneurysm Model

Three patients with intracranial bifurcation aneurysms, treated with stent-assisted coiling in real life, were included in this study. In case 1, a 66-year-old male with an unruptured small anterior communicating artery aneurysm (maximal diameter: 4.8 mm; width: 2.53 mm) was treated with a Neuroform (Stryker, Kalamazoo, Michigan, USA; size: 2.5 × 15mm) stent-assisted coiling embolization. For case 2, a 48-year-old female with a ruptured anterior communicating artery aneurysm (maximal diameter: 5.49 mm; width: 4.09 mm) was treated with LVIS (MicroVention, Tustin, CA, USA; size: 2.5 × 17mm) stent-assisted coiling. For case 3, a 57-year-old female with an unruptured A2/3 bifurcation aneurysm (maximal diameter: 6.96 mm; width: 7.22 mm) was treated with Solitaire AB (Covidien, Irvine, California; size: 4 × 20 mm) stent-assisted coiling embolization.

3D rotational angiographic images were obtained, while 3D segmentation and isolation of the region of interest were performed through the open-source software VMTK (www.vmtk.org). The segmented geometry before treatment is shown in Figure 1. To simplify the simulation of stenting, part of the adjacent parent artery with the aneurysm sac was isolated from the whole parent vessel using the Geomagic tool (Geomagic Inc., Morrisville, North Carolina). Our institutional review board approved this retrospective study with consent waived.

### Finite-Element Method Modeling of Stent Deployment

Solitaire, LVIS, and Neuroform stents were virtually generated using SolidWorks (Dassault Systems, SolidWorks Corp., MA) and transferred into FEM software ABAQUS v6.14 (SIMULIA, Providence, RI) to perform the remodeling of the aneurysm with adjacent parent vessels.

The FEM-based workflow for stent deployment modeling was conducted in ABAQUS/Explicit v6.14, where the stent was modeled as Nitinol alloy. The material properties were obtained from literature (9–11), as shown in Table 1. The simulation consists of three steps: crimping, delivery, and deployment. The crimping of the stent was performed and used for the initial condition for the delivery process using the predefined field tool in ABAQUS. The delivery path was generated with central points of cross-sections of the blood vessel. Crimped stent within the microcatheter was delivered through the path to the orifice of the aneurysm as the actual delivery process during clinical treatment. The crimped stent was assembled in a microcatheter in the global coordinate system and delivered to the aneurysm orifice of the pre-treatment model through a displacement load according to the central points of the arterial wall along the delivery path. The stent was released in the next step with the predefined stress-strain field. A “general contact” algorithm in ABAQUS was used for the complex interactions during the stent delivery and deployment procedures, with a friction coefficient value of 0.15 (12).

**Table 1:** Superelastic shape-memory alloys material properties for the Auricchio/Taylor superelasticity model (9–11).

**Thermoelastic properties**							
*E* ^*A*^		*E* ^*M*^		*v* ^*A*^		*v* ^*M*^	
70 GPa		70 GPa		0.33		0.33	
**Phase diagram properties**							
$\sigma^{M_{s}}$	${\sigma_{C}^{M_{s}}}^{\;}$	$\sigma^{M_{f}}$	$\sigma^{A_{s}}$	$\sigma^{A_{f}}$	*C* ^*A*^	*C* ^*M*^	*T*_0_
448 MPa	448 MPa	562 MPa	257 MPa	221 MPa	9.21 MPa/K	6.31 MPa/K	350 K
**Transformation strain properties**							
*H*				*H*_*V*_			
4.7%				4.7%			

**Figure 2:**
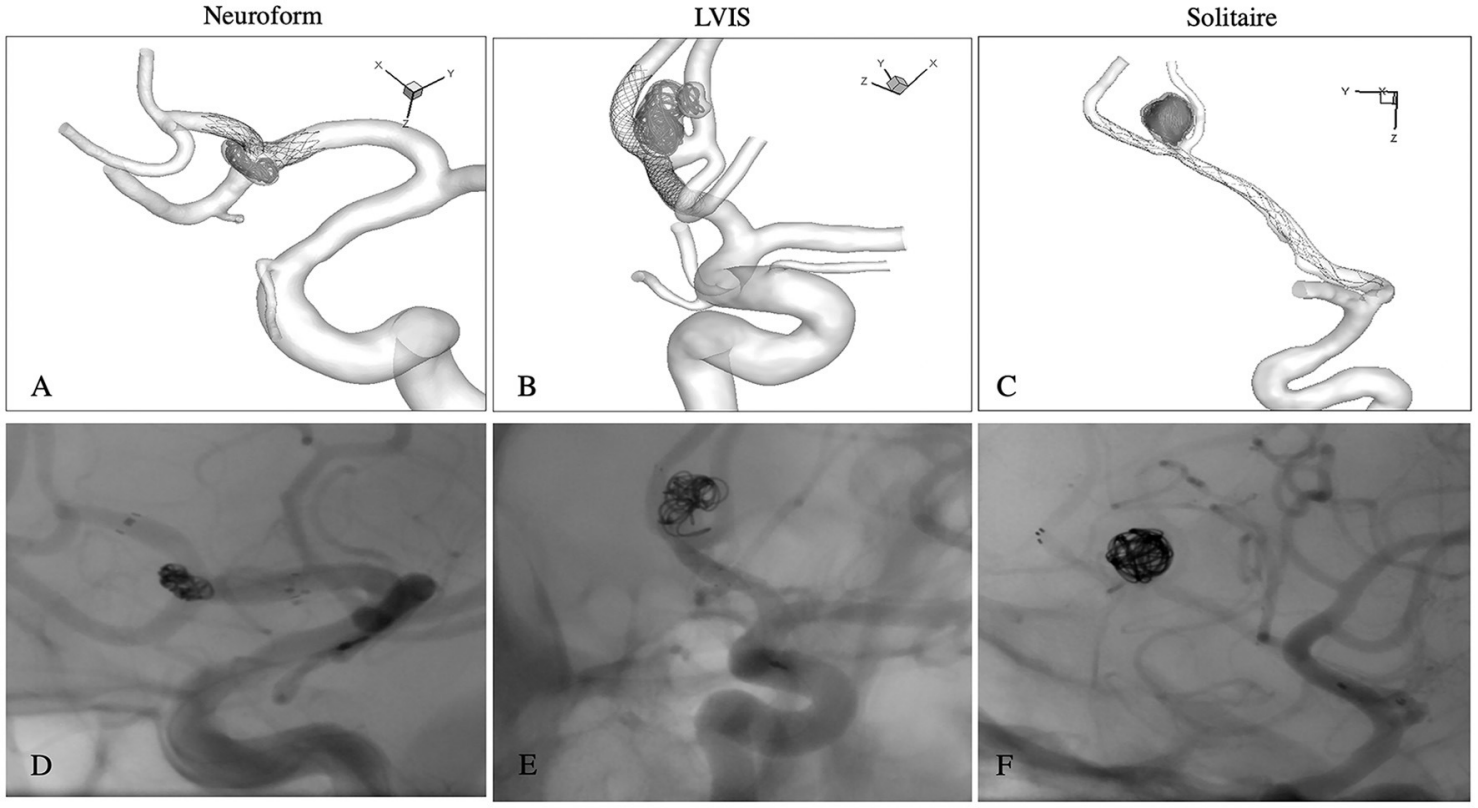
The FEM simulations of each stent matched the actual post-operative non-subtracted DSA images, including parent artery straightening. Neuroform stent **(A,D)**; LVIS stent **(B,E)**; Solitaire stent **(C,F)**.

During FEM analysis, the parent vessel was modeled as a rigid wall in no deformation models (model I and II) and deformable wall in straightened models (model III and IV). In the latter models, Mooney-Rivlin's stress-strain constitutive relationship was implemented to simulate the hyperplastic behavior of the vessel wall (13). A set of parameter values from the human cerebral artery wall were adopted, where the parameters were chosen as C1 = 0.174 MPa, C2 = 1.88 MPa (13). The cerebral arterial wall and aneurysmal wall were modeled as membrane elements with a thickness of 0.3 mm (14) and 0.2 mm (15), respectively. In the end, the surface-based aneurysm and vessel geometry model with the 3D representation of the stent were used for the subsequent CFD analysis. The exact method can be referenced in our previous study (7). The FEM simulations matched the actual stent-deployment images, including the parent artery straightening (Figure 2).

### CFD Simulation

Computational models meshed with polyhedral grids with a size of 0.1 mm for the aneurysm and vessel and 0.03 mm for the stent using STAR-CCM+ meshing tool (CD Adapco, Melville, NY). Incompressible Navier-Stokes equations under steady flow conditions were solved with the finite volume CFD solver, STAR-CCM+. The mean flow rate for the internal carotid artery inlet was 4.6 ml/s and this was used as the inlet boundary condition (16). Traction-free boundary conditions were applied at all outlets and, the mass flow rate through each outlet vessel was set to be proportional to the cube of its diameter based on the principle of optimal work (17). With a density of 1,056 kg/m^3^ and a viscosity of 0.0035 N·s/m^2^, the blood was modeled as a Newtonian fluid material (18), and the vessel walls were simulated as a rigid wall with no-slip boundary conditions (19).

**Figure 3:**
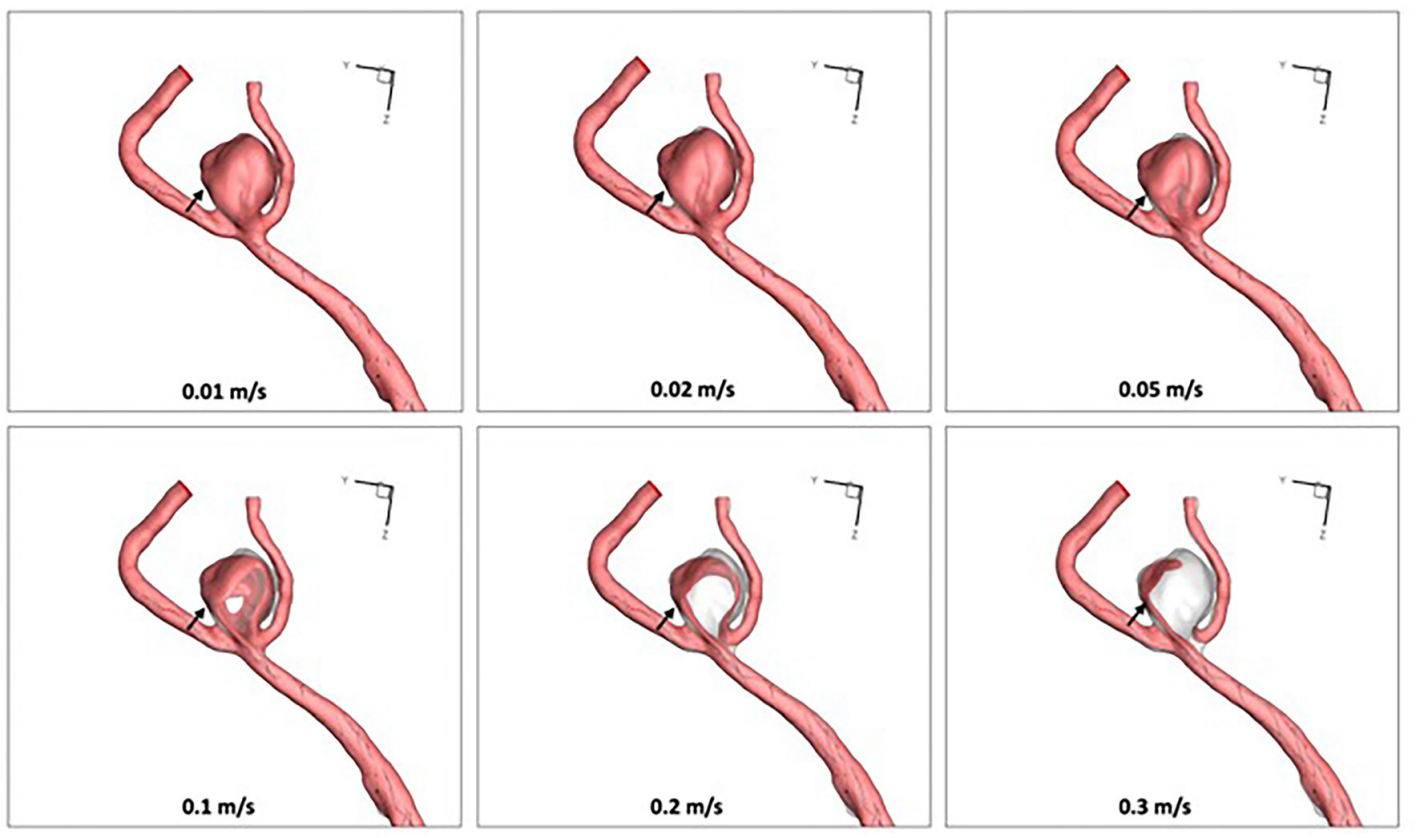
As the threshold velocity value increased in the virtual stenting with parent artery straigtening (Model IV), the high flow region (0.3 m/s) focused on the inflow trunk of the aneurysm neck (arrows).

*Bifurcation angle* was defined as the angle between the stented branch and the proximal main trunk of the aneurysm. The aneurysmal flow streamlines, iso-velocity surface (to measure high flow region around aneurysmal neck plane), and wall shear stress (WSS) were visualized for qualitative analysis. Iso-velocity surface was the surface with equal velocity. As the threshold value increased, the high flow region became focused on the aneurysm neck (Figure 3). In this study, the threshold value for velocity was set at 0.3 m/s. For quantitative analysis, the sac-averaged velocity, high flow volume using iso-velocity surface (>0.3 m/s), and sac-averaged WSS were calculated using the pre-treatment model as a baseline (100%).

## Results

### Bifurcation Angle Change

For the Neuroform stent model, the bifurcation angle change was 42.47° from pre-treatment 96.42° to post-stenting 138.89°. The aneurysm experienced no recurrence in the 20-month DSA follow-up. For the LVIS stent model, the bifurcation angle changed from 112.27 to 135.90° after stenting. Follow-up DSA after 30 months revealed no recurrence. The bifurcation change in the Solitaire stent model was most dramatic, from 58.5 to 168.27°, which almost became a side-wall aneurysm (Table 2). The aneurysm was not recurrent in the 10-month DSA follow-up.

**Table 2:** Angular measurements and hemodynamics of four models in three different stents.

	**Bifurcation angle**	**WSS**	**Velocity**	**High flow volume**
	**(degrees)**	**(Pa)**	**(m/s)**	**(mm3)**
**Neuroform**
Model I	96.42	4.30 (100%)	0.180 (100%)	3.23 (100%)
Model II	96.42	3.54 (82.3%)	0.159 (88.3%)	2.51 (77.7%)
Model III	138.89	3.07 (71.4%)	0.134 (74.4%)	1.42 (44.0%)
Model IV	138.89	2.45 (57.0%)	0.113 (62.8%)	0.617 (19.1%)
**LVIS**
Model I	112.27	12.72 (100%)	0.434 (100%)	51.0 (100%)
Model II	112.27	13.36 (105.0%)	0.396 (91.2%)	46.90 (92.0%)
Model III	135.90	5.11 (40.2%)	0.252 (58.1%)	28.60 (56.1%)
Model IV	135.90	5.06 (39.8%)	0.228 (52.5%)	22.4 (43.9%)
**Solitaire**
Model I	58.50	9.70 (100%)	0.292 (100%)	64.2 (100%)
Model II	58.50	10.25 (105.7%)	0.325 (111.3%)	74.20 (115.6%)
Model III	168.27	4.13 (42.6%)	0.136 (46.6%)	9.67 (15.1%)
Model IV	168.27	3.82 (39.4%)	0.125 (42.8%)	8.70 (13.6%)

### Qualitative Analysis

**Figure 4:**
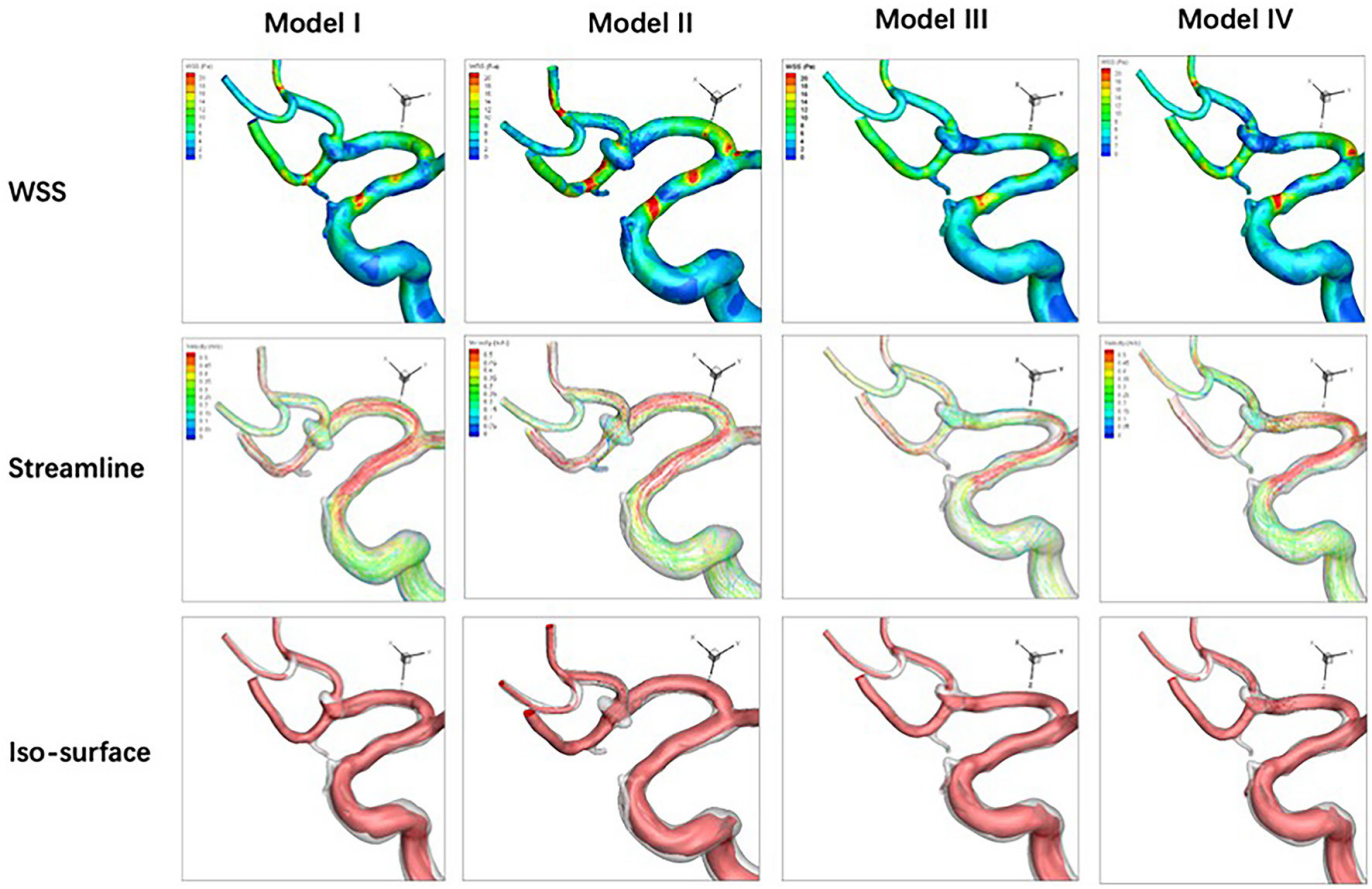
For an anterior communicating artery aneurysm with Neuroform stenting, hemodynamic characteristics of four models are depicted. Wall shear stress, velocity, and high flow volume in Models III and IV decrease slightly compared with Models I and II.

**Figure 5:**
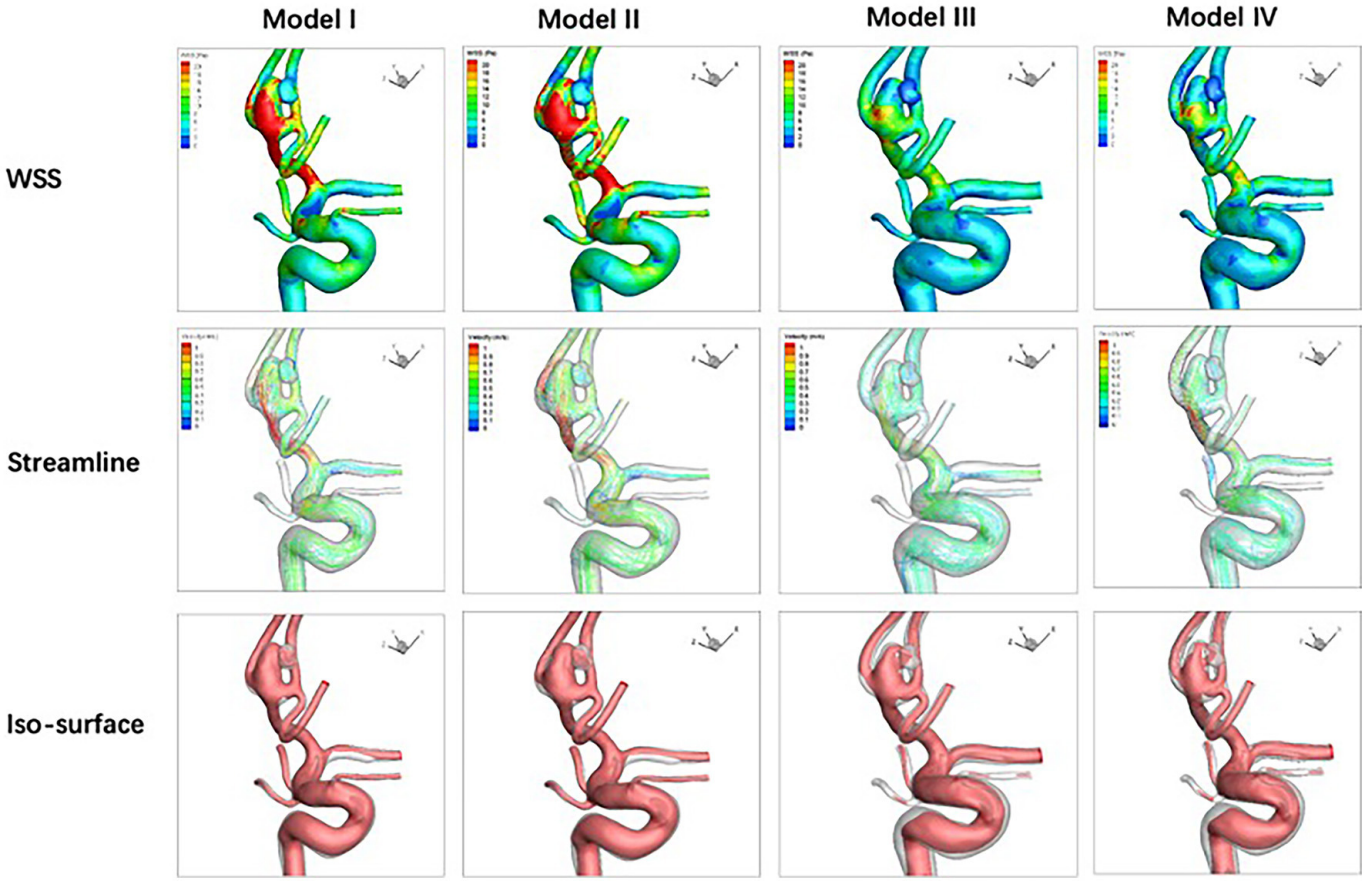
Hemodynamic characteristics of four models are illustrated in LVIS stenting for an anterior communicating artery aneurysm with a daughter sac. Compared with Model I, wall shear stress increases, and velocity decreases while the high flow volume does not change significantly in Model II. Wall shear stress, velocity, and high flow volume in Models III and IV decreased significantly compared with Model I.

**Figure 6:**
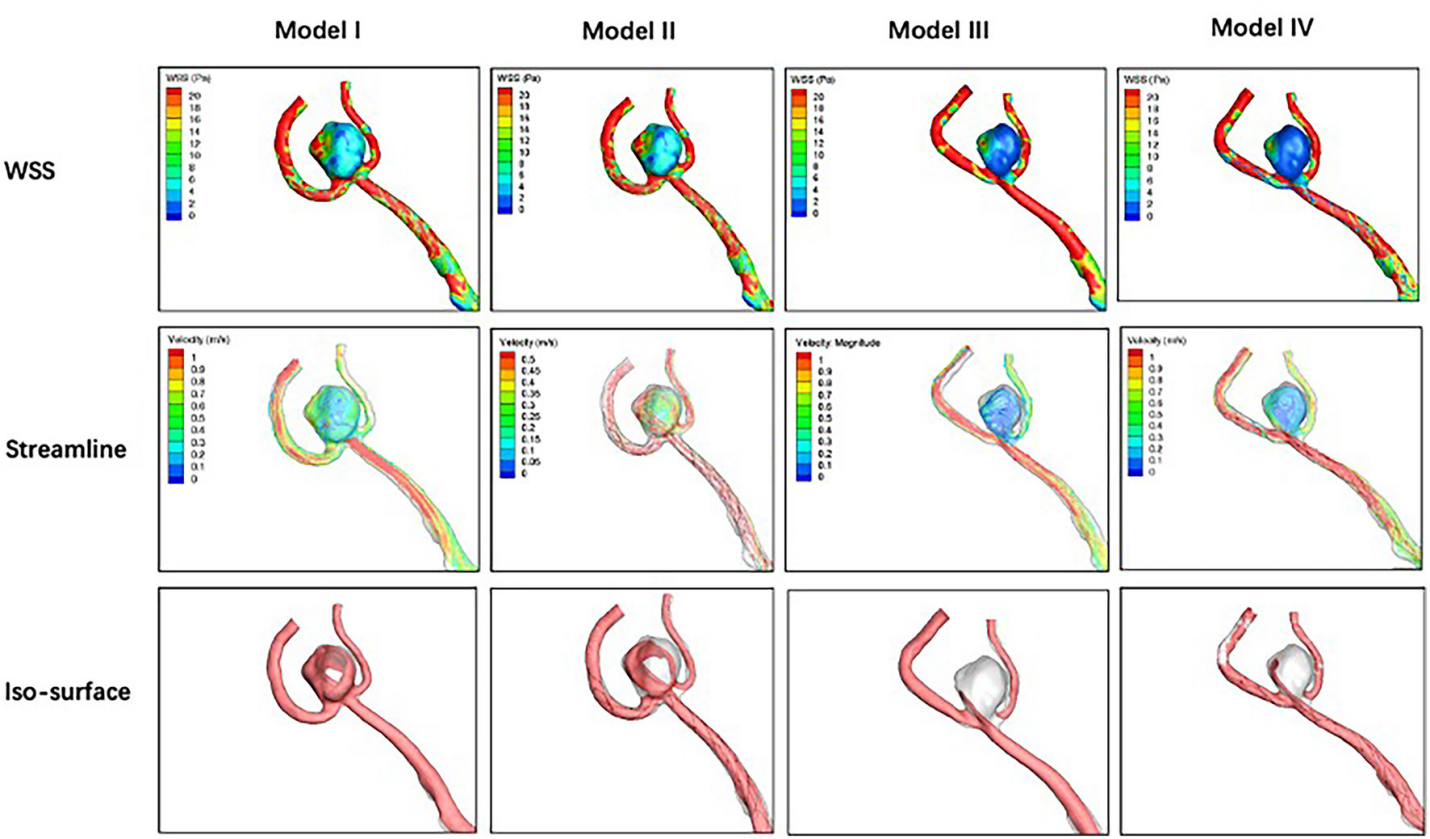
Hemodynamic characteristics of four models for Solitaire stenting of an unruptured A2/3 bifurcation aneurysm are revealed. Velocity in Model II increased significantly while wall shear stress and high flow volume in Model II do not change significantly compared with Model I. Wall shear stress, velocity, and high flow volume in Models III and IV decreased significantly compared with Model I.

Compared with the pre-treatment baseline (Model I), stenting with parent artery reconstruction (Model IV) in the three stents performed the best in decreasing mean WSS, velocity, and high flow volume. In LVIS and Solitaire stent groups, the WSS of stenting without parent artery reconstruction (Model II) increased compared with corresponding pre-treatment models. In the Solitaire stent, the velocity and high flow volume of Model II increased compared with Model I. In three stent groups, the WSS, velocity, and high flow volume of Model III were lower than these of Model II (Figures 4-6).

### Quantitative Analysis

**Figure 7:**
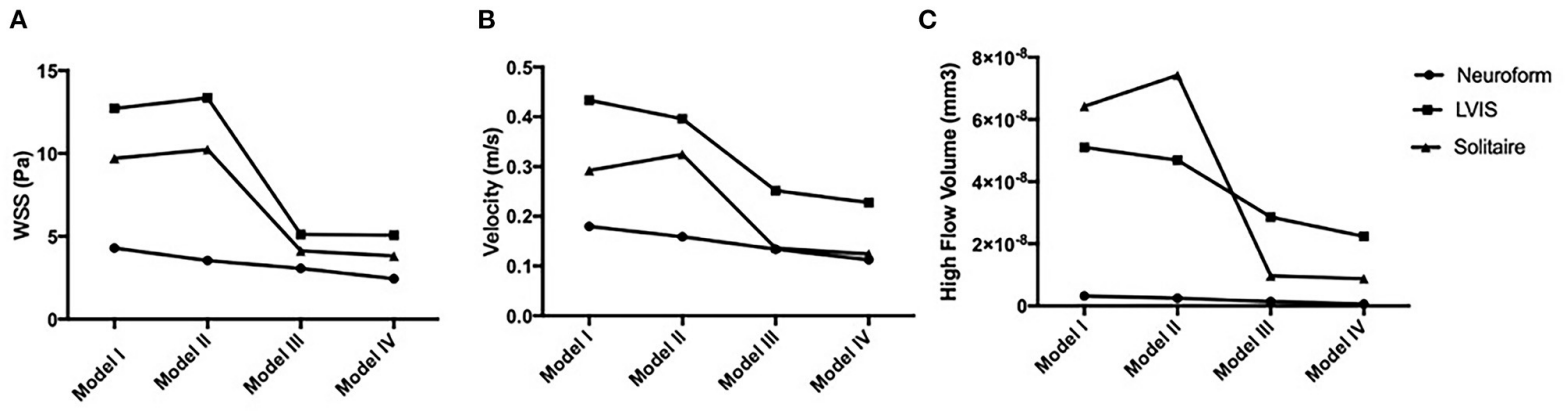
Hemodynamic characteristics tendencies for the four models in three stents are analyzed. **(A)** Wall shear stress (WSS); **(B)** velocity (m/s); **(C)** high flow volume (mm3).

For the Neuroform stent, compared with the pre-treatment model (100%), the mean WSS decreased to 82.3, 71.4, and 57.0% in models II-IV, velocity to 88.3, 74.4, and 62.8%, and HFV to 77.7, 44.0, and 19.1%. For the LVIS stent, the mean WSS changed to 105.0, 40.2, and 39.8% in models II-IV, velocity to 91.2, 58.1, and 52.5%, and HFV to 92.0, 56.1, and 43.9%. With the Solitaire stent, compared with the pre-treatment model (100%), the mean wall shear stress (WSS) of Models II-IV changed to 105.7, 42.6, and 39.4%, the sac-averaged velocity changed by 111.3, 46.6, and 42.8%, and the high flow volume (HFV, >0.3m/s) changed by 115.6, 15.1, and 13.6% (Figure 7).

## Discussion

This study simulated four models for each of three different stents to reveal the hemodynamics induced by the stent straightening effect. The hemodynamic effects of stent-induced parent artery straightening are better than the stent-mesh effect in different stent models. Hypothesized stenting without a parent artery straightening model could produce adverse effects. Fortunately, the actual stenting with the parent artery straighening model plays the best performance in modifying the aneurysm hemodynamics.

For the initiation of intracranial aneurysms, bifurcation angulation plays an important role (20–22), with Song et al. finding that a larger bifurcation angle was more prevalent on the aneurysmal branch compared with the contralateral non-aneurysmal middle cerebral artery bifurcation (23). Furthermore, intracranial aneurysm presence was associated with abnormal hemodynamics due to the abnormal bifurcation angle (23, 24). The stent-induced parent artery straightening concept has been increasingly adopted clinically to transform a bifurcation aneurysm into a sidewall aneurysm (25, 26). Stent-induced straightening of the parent artery can decrease recanalization, especially for intracranial bifurcation aneurysms (4, 26). In computational fluid dynamics, Gao et al. (6) revealed that stent-induced angular remodeling significantly altered bifurcation apex hemodynamics in a favorable direction and narrowed and migrated the flow impingement zone based on aneurysm-capped simulation. In our study, stenting after parent artery reconstruction (Model IV) directly confirmed that straightening the parent artery decreased the wall shear stress and velocity and migrate the high flow region in the aneurysm neck. Neointima formation and thrombus organization are concurrent processes during aneurysm healing (27). Conventional stents work as a scaffold for neointima formation (28). However, stent-induced straightening of the parent artery can decrease the high flow volume in the aneurysm sac, facilitating thrombus in the aneurysm sac.

Previous studies revealed that LVIS close mesh could divert flow and double LVIS induced flow diverter effect could surpass a Pipeline stent (8), although the unpredictability in overlapping stent use. In this study, the Solitaire stent-mesh size is the largest (29), indicating its weakest strut effect. However, the Solitaire stent straightened the parent artery significantly and transformed the bifurcation aneurysm into a sidewall one. The hemodynamic straightening effect increased while the parent artery angle changes increased. This study did not simulate a Pipeline stent and compared its flow diverter hemodynamic effect with straightening parent artery due to its off-label application in intracranial bifurcation aneurysms. Clinically, flow diverter embolization devices have been used in complex bifurcation aneurysms beyond the circle of Willis in some centers. However, the branch caliber reduction and asymptomatic occlusion of covered cortical branches and silent perforator stroke are not uncommon (30).

For sidewall aneurysms, a previous study demonstrated that stent struts had a dual effect on flow velocity reduction than straightening vessels (31). In contrast, our study revealed that the hypothesized stenting without straightening could generate an adverse hemodynamic impact in the bifurcation aneurysms. We theorized that the stent struts could narrow the inflow jet typically observed in bifurcation IAs and generate elevated flow inside the aneurysm sac. Jeong et al. (5) also found an adverse effect due to stenting of bifurcation aneurysms.

WSS indicates the frictional force between blood and arterial wall inner surface and can influence aneurysm initiation with high WSS (32), and ruptures with low WSS (33). A low aneurysmal WSS environment encourages inflammatory cell infiltration and has been correlated with aneurysm rupture status. Stagnant flow and excessively low WSS after stenting or flow diverter may induce focal inflammation and subsequent tissue destruction or degradation in the aneurysm dome, the usual rupture site. Low WSS accelerates unstable red thrombus formation, while high WSS facilitates stable white thrombus after stenting. The three aneurysms in this study were treated with stent-assisted coiling. Coiling may facilitate thrombus formation in the aneurysm sac before aneurysm wall degradation and rupture.

Some limitations must be noted. First, the sample is small, which needs further extensive sample studies to demonstrate. Second, we adopted several commonly used assumptions to make CFD tractable. Due to a lack of patient-specific information, we assumed a constant, location-based inlet flow rate. Inlet velocities were scaled according to the inlet diameter. This study utilized the pretreatment model as a baseline and evaluated the relative, not absolute hemodynamic change. Future studies should consider utilizing a pulsatile flow profile instead of steady-state to explore the detailed effect of vessel straightening and its impact on hemodynamics within the aneurysm.

## Conclusion

The hemodynamic effect of straightening the parent artery induced by stenting was markedly better than that of stent mesh flow diversion in all three different stents tested. Stent-induced remodeling of the parent artery, transforming the bifurcation aneurysms into sidewall aneurysms, should decrease the recurrence rate in intracranial bifurcation aneurysms.

## Data Availability Statement

The raw data supporting the conclusions of this article will be made available by the authors, without undue reservation.

## Ethics Statement

The studies involving human participants were reviewed and approved by Huashan Hospital, Fudan University. The patients/participants provided their written informed consent to participate in this study.

## Author Contributions

XZ and JX had the idea for the article. HW and XL performed the computational fluid study. HW, LH, GL, LG, and YJ performed the literature search. HW and LH wrote the article. XZ and JX are the guarantors. All authors contributed to the article and approved the submitted version.

## Funding

This study was supported by National Nature Science Foundation of China (Grant No. 81771242) and National Nature Science Foundation of China (Grant No. 81371308).

## Conflict of Interest

XL and JX were employed by the company ArteryFlow Technology Co., Ltd. The remaining authors declare that the research was conducted in the absence of any commercial or financial relationships that could be construed as a potential conflict of interest.

## Publisher's Note

All claims expressed in this article are solely those of the authors and do not necessarily represent those of their affiliated organizations, or those of the publisher, the editors and the reviewers. Any product that may be evaluated in this article, or claim that may be made by its manufacturer, is not guaranteed or endorsed by the publisher.

## References

[1] Zhang X Zuo Q Tang H Xue G Yang P Zhao R. Stent assisted coiling versus non-stent assisted coiling for the management of ruptured intracranial aneurysms: a meta-analysis and systematic review. J NeuroInterv Surg. (2019) 11:489–96. 10.1136/neurintsurg-2018-014388 30842307

[2] Huang QH Wu YF Xu Y Hong B Zhang L Liu JM. Vascular geometry change because of endovascular stent placement for anterior communicating artery aneurysms. Am J Neuroradiol. (2011) 32:1721. 10.3174/ajnr.A2597 21816920 PMC7965400

[3] Gao B Baharoglu MI Cohen AD Malek AM. Y-stent coiling of basilar bifurcation aneurysms induces a dynamic angular vascular remodeling with alteration of the apical wall shear stress pattern. Neurosurgery. (2013) 72:617–29. 10.1227/NEU.0b013e3182846d9f 23277371

[4] Ishii A Chihara H Kikuchi T Arai D Ikeda H Miyamoto S. Contribution of the straightening effect of the parent artery to decreased recanalization in stent-assisted coiling of large aneurysms. J Neurosurg. (2017) 127:1063–9. 10.3171/2016.9.JNS16501 28009233

[5] Jeong W Han MH Rhee K. The hemodynamic alterations induced by the vascular angular deformation in stent-assisted coiling of bifurcation aneurysms. Comput Biol Med. (2014) 53:1–8. 10.1016/j.compbiomed.2014.07.006 25089357

[6] Gao B Baharoglu MI Malek AM. Angular remodeling in single stent-assisted coiling displaces and attenuates the flow impingement zone at the neck of intracranial bifurcation aneurysms. Neurosurgery. (2013) 72:739–48; discussion 748. 10.1227/NEU.0b013e318286fab3 23328687

[7] Leng X Wan H Li G Jiang Y Huang L Siddiqui AH. Hemodynamic effects of intracranial aneurysms from stent-induced straightening of parent vessels by stent-assisted coiling embolization. Interv Neuroradiol. (2021) 27:181–90. 10.1177/1591019921995334 33641496 PMC8050531

[8] Wang C Tian Z Liu J Jing L Paliwal N Wang S. Flow diverter effect of lvis stent on cerebral aneurysm hemodynamics: a comparison with enterprise stents and the pipeline device. J Transl Med. (2016) 14:199. 10.1186/s12967-016-0959-9 27370946 PMC4930570

[9] Reedlunn B Daly S Shaw J. Tension-torsion experiments on superelastic shape memory alloy tubes. In: ASME 2012 Conference on Smart Materials, Adaptive Structures and Intelligent Systems. (2012) 2012:213–22. 10.1115/SMASIS2012-8185

[10] Zhu P Brinson LC Peraza-Hernandez E Hartl D Stebner A. Comparison of three-dimensional shape memory alloy constitutive models: finite element analysis of actuation and superelastic responses of a shape memory alloy tube. In: ASME 2013 Conference on Smart Materials, Adaptive Structures and Intelligent Systems. (2013) 2013:V002T002A004. 10.1115/SMASIS2013-3093

[11] Leng X Wang Y Xu J Jiang Y Zhang X Xiang J. Numerical simulation of patient-specific endovascular stenting and coiling for intracranial aneurysm surgical planning. J Transl Med. (2018) 16:208. 10.1186/s12967-018-1573-9 30031395 PMC6054731

[12] Damiano R Ma D Xiang J Siddiqui AH Snyder KV Meng H. Finite element modeling of endovascular coiling and flow diversion enables hemodynamic prediction of complex treatment strategies for intracranial aneurysm. J Biomech. (2015) 48:3332–40. 10.1016/j.jbiomech.2015.06.018 26169778 PMC4801175

[13] Zhang H Jiao Y Johnson E Zhan L Zhang Y Shimada K. Modelling anisotropic material property of cerebral aneurysms for fluid–structure interaction simulation. Comput Methods Biomech Biomed Eng Imaging Visual. (2013) 1:164–74. 10.1080/21681163.2013.776270

[14] Tóth M Nádasy GL Nyáry I Kerényi T Monos E. Are there systemic changes in the arterial biomechanics of intracranial aneurysm patients? Pflügers Archiv. (2000) 439:573–8. 10.1007/s004249900154 10764217

[15] Eriksson T Kroon M Holzapfel GA. Influence of medial collagen organization and axial in situ stretch on saccular cerebral aneurysm growth. J Biomech Eng. (2009) 131:101010–7. 10.1115/1.3200911 19831480

[16] Fahrig R Nikolov H Fox AJ Holdsworth DW. A three-dimensional cerebrovascular flow phantom. Med Phy. (1999) 26:1589–99. 10.1118/1.598672 10501059

[17] Oka S Nakai M. Optimality principle in vascular bifurcation. Biorheology. (1987) 24:737–51. 10.3233/BIR-1987-24624 3502768

[18] Xiang J Yu J Snyder KV Levy EI Siddiqui AH Meng H. Hemodynamic–morphological discriminant models for intracranial aneurysm rupture remain stable with increasing sample size. J NeuroInterv Surg. (2016) 8:104. 10.1136/neurintsurg-2014-011477 25488922 PMC4791310

[19] Cebral JR Castro MA Appanaboyina S Putman CM Millan D Frangi AF. Efficient pipeline for image-based patient-specific analysis of cerebral aneurysm hemodynamics: technique and sensitivity. IEEE Trans Med Imaging. (2005) 24:457–67. 10.1109/TMI.2005.844159 15822804

[20] Baharoglu MI Lauric A Safain MG Hippelheuser J Wu C Malek AM. Widening and high inclination of the middle cerebral artery bifurcation are associated with presence of aneurysms. Stroke. (2014) 45:2649–55. 10.1161/STROKEAHA.114.005393 25116869 PMC4146742

[21] Zhang XJ Gao BL Hao WL Wu SS Zhang DH. Presence of anterior communicating artery aneurysm is associated with age, bifurcation angle, and vessel diameter. Stroke. (2018) 49:341–7. 10.1161/STROKEAHA.117.019701 29301972

[22] Tutuncu F Schimansky S Baharoglu MI Gao B Calnan D Hippelheuser J. Widening of the basilar bifurcation angle: association with presence of intracranial aneurysm, age, and female sex. J Neurosurg. (2014) 121:1401–10. 10.3171/2014.8.JNS1447 25280096

[23] Song J Zhu F Qian Y Ou C Cai J Zou X. Morphological and hemodynamic differences between aneurysmal middle cerebral artery bifurcation and contralateral nonaneurysmal anatomy. Neurosurgery. (2017) 81:779–86. 10.1093/neuros/nyx093 28379506

[24] Zhang XJ Li CH Hao WL Zhang DH Ren CF Gao BL. Enlarged anterior cerebral artery bifurcation angles may induce abnormally enhanced hemodynamic stresses to initiate aneurysms. World Neurosurg. (2018) 120:e783–91. 10.1016/j.wneu.2018.08.167 30176397

[25] Adeeb N Griessenauer CJ Patel AS Foreman PM Baccin CE Moore JM. The use of single stent-assisted coiling in treatment of bifurcation aneurysms: a multicenter cohort study with proposal of a scoring system to predict complete occlusion. Neurosurgery. (2018) 82:710–8. 10.1093/neuros/nyx310 28595331

[26] Funakoshi Y Imamura H Tani S Adachi H Fukumitsu R Sunohara T. Effect of straightening the parent vessels in stent-assisted coil embolization for anterior communicating artery aneurysms. World Neurosurg. (2019) 126:e410–6. 10.1016/j.wneu.2019.02.066 30822575

[27] Gruter BE Wanderer S Strange F Boillat G Taschler D Rey J. Patterns of neointima formation after coil or stent treatment in a rat saccular sidewall aneurysm model. Stroke. (2021) 52:1043–52. 10.1161/STROKEAHA.120.032255 33504186

[28] Marbacher S Niemela M Hernesniemi J Frosen J. Recurrence of endovascularly and microsurgically treated intracranial aneurysms-review of the putative role of aneurysm wall biology. Neurosurg Rev. (2019) 42:49–58. 10.1007/s10143-017-0892-2 28819834

[29] Krischek Ö Miloslavski E Fischer S Shrivastava S Henkes H. A comparison of functional and physical properties of self-expanding intracranial stents [neuroform3, wingspan, solitaire, leo(+), enterprise]. Minim Invasive Neurosurg. (2011) 54:21–8. 10.1055/s-0031-1271681 21506064

[30] Michelozzi C Darcourt J Guenego A Januel AC Tall P Gawlitza M. Flow diversion treatment of complex bifurcation aneurysms beyond the circle of willis: Complications, aneurysm sac occlusion, reabsorption, recurrence, and jailed branch modification at follow-up. J Neurosurg. (2018) 131:1751–62. 10.3171/2018.7.JNS18654 30579280

[31] Kono K Shintani A Terada T. Hemodynamic effects of stent struts versus straightening of vessels in stent-assisted coil embolization for sidewall cerebral aneurysms. PLoS ONE. (2014) 9:e108033. 10.1371/journal.pone.0108033 25247794 PMC4172595

[32] Kulcsár Z Ugron Á Marosfoi M Berentei Z Paál G Szikora I. Hemodynamics of cerebral aneurysm initiation: The role of wall shear stress and spatial wall shear stress gradient. Am J Neuroradiol. (2011) 32:587–94. 10.3174/ajnr.A2339 21310860 PMC8013095

[33] Meng H Tutino VM Xiang J Siddiqui A. High wss or low wss? Complex interactions of hemodynamics with intracranial aneurysm initiation, growth, and rupture: toward a unifying hypothesis. Am J Neuroradiol. (2014) 35:1254–62. 10.3174/ajnr.A3558 23598838 PMC7966576

